# Benefits and challenges in the use of RE-AIM for evaluation of a national social work staffing program in the veterans health administration

**DOI:** 10.3389/frhs.2023.1225829

**Published:** 2023-11-16

**Authors:** Portia Y. Cornell, Cassandra L. Hua, Christopher W. Halladay, Jaime Halaszynski, Alita Harmon, Jennifer Koget, Jennifer W. Silva

**Affiliations:** ^1^ Providence Veterans Affairs (VA) Medical Center, Center of Innovation for Long Term Services and Supports, Providence, RI, United States; ^2^ Department of Health Services, Policy, and Practice, Brown University School of Public Health, Providence, RI, United States; ^3^ Butler VA Health Care System, Butler, PA, United States; ^4^ Department of Veterans Affairs, Veterans Health Administration, Office of Care Management and Social Work Services, National Social Work Program, Washington, DC, United States; ^5^ Gulf Coast Veterans Health Care System, Biloxi, MS, United States

**Keywords:** social work, RE-AIM, program evaluation, implementation, social determinants of health, Veterans

## Abstract

**Background:**

In the Department of Veterans Affairs (VA) Veterans Health Administration (VHA), social workers embedded in primary care teams address social and emotional needs that are associated with health outcomes. The mission of the National Social Work PACT Staffing Program is to improve access to social work services for rural Veterans by supporting additional social work staffing in VA medical centers serving rural areas.

**Methods:**

We obtained data from the VA corporate data warehouse on Veterans’ characteristics and health care use from 2016 to 2022 for all Veterans who received primary care at a Veterans Affairs Medical Center (VAMC) or associated clinic that received funding from the program. We evaluated the program according to RE-AIM constructs as follows: Reach [total number of Veterans who engaged with PACT social work and representativeness with regard to race, rural residence, chronic conditions and health behaviors, and hospital and emergency department (ED) use in the previous 12 months]; Effectiveness (impact of the program on key health care use outcomes which include hospitalizations, emergency department visits, and palliative care); Adoption (number of VA medical centers and outpatient clinics serving rural Veterans that have participated in the program, and number and representativeness of sites eligible for program participation that have not yet received funding); Implementation (adherence to standardized note templates), and Maintenance (permanent social work positions created by the program and continued technical support).

**Results:**

In 2022, the program engaged with 30,982 Veterans, 65% of whom lived in rural areas. The program increased social work encounters, reduce hospital and emergency department use, and increase use of palliative care services among Veterans. Key elements of implementation include proactive outreach to Veterans with high-risk indicators and assessment for social risk factors using standardized, national note templates. In terms of maintenance, the program continues to provide data and technical assistance to 23 sites and has created 171 permanent social work positions.

**Conclusions and implications:**

The Social Work PACT Staffing Program demonstrates positive outcomes and program sustainment. The RE-AIM framework was a useful tool to evaluate the program, but additional adaption was needed to fit the program’s needs.

## 1. Introduction

Social workers in health care settings play a key role in the provision of primary care; they can act as care coordinators, educate individuals about resources, provide counseling, assess for social risks and needs, and serve as advocates for patients. In 2010, VA reorganized the delivery of primary care around the concept of primary care medical homes, now referred to as Patient Aligned Care Teams (PACTs). PACTs use the “teamlet” approach which consists of one Primary Care Provider, a Registered Nurse, a Clinical Associate and an Administrative Associate, with an assigned a panel of approximately 1,200 Veterans (1). A fully implemented PACT has one licensed master's-prepared social worker (2) per two fully staffed primary care teamlets, and a ratio of one social worker to 2,400 Veterans (3). This social worker provides social work services to all Veterans in their panel on an as-needed basis, including those with complex health, mental health, and social needs.

In 2014, staffing of social workers in the PACT model remained inconsistent. Frequently, PACTs serving rural populations lacked social work coverage, creating service gaps for Veterans. In 2016, the VA National Social Work Program in Care Management and Social Work Services, in collaboration with the Office of Rural Health, implemented the VA Social Work in Patient Aligned Care Teams (PACT) Staffing Program. This enterprise-wide initiative provided 3-year seed funding for additional PACT social workers at sites serving rural and highly rural Veterans. These sites included participating outpatient clinics co-located in a medical center as well as stand-alone, community-based outpatient clinics. Broadly, the interventions that PACT social workers provide address chronic social and healthcare needs to prevent acute health problems. Social workers address health-related social needs that can increase allostatic load through stress, exacerbate existing health problems, and act as barriers to appointments and appropriate preventive and primary care (4–6). Social workers were trained to use the VA's Social Work Practice Model, which emphasizes comprehensive assessment of social needs in six core social determinant of health domains—access to care, economics, housing, social support, psychological status, functional status—followed by intervention and ongoing support.

The RE-AIM framework is commonly used to frame evaluations, encompassing Reach (participation rate and representativeness), Effectiveness (impact on outcomes), Adoption (proportion and representativeness of site participation), Implementation (consistency of delivery by settings and implementation strategies), and Maintenance (extent intervention becomes part of routine practice). The RE-AIM framework has been used to evaluate social work interventions (7–9). Thus far and to our knowledge, there is scant published literature on the use RE-AIM to guide social-work interventions of national scope, and none in the VA context. With the growing attention to the need to measure and address social determinants of health in the health care system, we expect that program planners will have an increasing need for tools to support the design and evaluation of centered on Veterans' social needs.

Application of RE-AIM is ideal when the goal of program evaluation extends beyond efficacy and information is needed regarding how well an intervention is integrated into real-world settings, especially for complex interventions. In this context, for example, Reach is important to understand given that the focus of the Office of Rural Health, in supporting the program, aimed to serve rural Veterans. Additionally, it is critical to understand whether the Social Work PACT Staffing Program is integrated into routine practice, as well as adoptions needed to sustain staffing levels, because funding is concluded at the end of the intervention (10).

The objective of this study was to evaluate the Social Work PACT Staffing Program using the RE-AIM framework. In addition to providing an evaluation of the program, we discuss benefits and limitations of using the RE-AIM model as a guiding framework.

## 2. Methods

This evaluation was done as part of quality improvement activities on behalf of Care Management and Social Work Services and determined exempt from review by the Providence VA Medical Center institutional review board.

### 2.1. Intervention

#### 2.1.1. The social work practice model

During all phases of program participation, PACT social workers are provided training in the VA Social Work Practice Model, including specific utilization of three required national note templates (i.e., the Social Work Triage Assessment, Social Work Comprehensive Assessment and Social Work Case Management note templates). The National Social Work PACT Staffing Program also offers monthly open office hours calls in addition to community-of-practice calls to provide additional support and to encourage resource sharing across the VA PACT Social Work community.

The PACT Social Work Practice Model guides VA PACT social workers' activities and interventions. PACT social workers are integrated into the PACT teamlet, and serve as a facilitator, advocate, and educator for the Veteran, family, and/or caregiver, and the interdisciplinary team. Short-term interventions by social workers might include advance care planning; referring the Veteran with the Veterans Benefits Administration or other VA resources for financial, education, or other disability programs; or community partners like Veterans Service Organizations for social, employment, or disability programs. Social workers may also provide longer-term case management by documenting a Veteran's healthcare preferences and providing ongoing counseling in support of the Veteran's health-related social needs and goals. Finally, PACT social workers play an important role in the VA in identifying mental health problems, with a referral or warm handoff to behavioral health or substance use services and providing evidence-based treatments such as motivational interviewing. They support the integration of mental health within primary care.

The Social Work PACT Staffing Program has five phases: Spark, Seed, Sprout, Sustain and Spread, developed from the VA's “innovation ecosystem” model (11). During the Spark phase of program participation, the National Social Work PACT Staffing Program leadership team actively recruits sites who identify barriers in serving rural or highly rural Veterans within their site's catchment areas.

The Seed phase begins once a site has been selected and approved for funding and they remain in this phase during their first year. Sites are provided full program orientation and are given direct hire authority. Sites are required to attend monthly Champions calls where key program information and data are shared and to offer a place for networking with other sites. National program leadership provides oversight and guidance around program metrics, budgeting, hiring, and training.

Sprout sites have moved into their second or third year of funding and continue to participate in programming as outlined for Seed, these sites are often asked to test and support program enhancements.

Sustainment phase sites have completed the funded portion of the program and have successfully implemented the VA Social Work Practice Model. A long-term plan is developed to ensure staffing sustainability. Sustainment sites participate in quarterly champions calls, provide monthly staffing reports for data collection and reporting, and attend trainings and receive consultation. Though funding has concluded, sites remain an active component during this and the next phase, Spread. These sites are often looked to for testing and piloting new intervention or ideas to enhance services for rural Veterans.

Spread is the final phase which sustains the VA Social Work Practice Model as the standard of practice and care at the site. Once a sustainment site has reached spread, monitoring and provision of customer service continue to ensure that rural Veterans are served after funding concludes.

### 2.2. Data and sample

#### 2.2.1. Program staffing

Program leadership maintained a list of names of social workers in actively funded and sustainment positions and the dates that they were in a program-funded position. A site was considered “active” or “sustainment” when it had a social worker assigned to the funded or previously funded position, respectively. The staffing list was used to precisely identify intervention start dates and social work encounters by funded positions.

Data on total staffing ratios for all VA medical centers were collected as part of a national assessment by Care Management and Social Work Services. A questionnaire was sent on behalf of the National Social Work Program to social work Chiefs and Executives. The questionnaire elicited total social work staff full-time equivalent (FTE) positions assigned to PACT.

#### 2.2.2. Veteran population

We used Veterans' data from the VA Corporate Data Warehouse, the national database of medical records for individuals enrolled in the VA health system. We included all Veterans who had one or more primary care encounters at a site that participated in the Social Work PACT Staffing Program. Social work encounters and national notes templates were identified using social work stop codes and text matches with notes templates (see Appendix). We identified encounters with funded social workers by matching the encounter with the social work staffing list. Encounter data were included for fiscal year (FY) 2022, from 1 October 2021 to 30 September 2022, and diagnosis codes, hospitalizations, and emergency department (ED) visits 12 months prior to the first encounter. The section on the “effectiveness” domain describes previously published analysis from the start of the program in FY 2016.

### 2.3. Analyses: Re-AIM constructs

In Reach analyses, Veteran characteristics were compared using 2-sided *t*-tests. Effectiveness analyses used difference-in-difference models that leveraged the expansion of the program over time so that sites that entered the program in later years served as a comparison group for sites that entered the program earlier. The effectiveness measures evaluated were number of Veterans in a month with any social work engagement, hospitalizations, emergency department use, and palliative care use. The methods are explained in detail in previously published work (12, 13). For the “adoption” domain, sites were designated as “eligible” for the Staffing Program if more than 50% of Veterans in primary care lived in rural or highly rural areas based on ZIP codes, or if more than 50% Veterans engaged with social work are from rural or highly rural areas. Implementation was measured by the utilization of national notes templates. Staffing ratios over time were used to measure Maintenance. These were calculated by site and were determined by dividing the total FTE social workers assigned to PACT, divided by the number of unique Veterans who received primary care in the fiscal year. Statistical analyses were performed in R (version X64 4.1.2).

## 3. Results

### 3.1. Reach

In FY 2022, social workers at Seed, Sprout or Sustainment sites within the Social Work PACT Staffing Program engaged with 30,892 unique Veterans (20,182 in rural or highly rural areas). This included 15,269 Veterans who engaged with social workers in currently funded positions across 21 sites (10,914 in rural or highly rural areas).

Care for this population of Veterans is represented by 50,798 total PACT social work encounters by social workers at currently or previously funded sites, including 25,605 encounters with currently funded social-work positions and 18,812 encounters with Veterans in rural and highly rural areas.

There were 507,329 Veterans identified who had at least one primary care visit at a Seed, Sprout or Sustainment site in FY 2022. Among these, 43,720 (8.6%) Veterans engaged with a PACT social worker at least once during the year. In Table 1 characteristics of the two groups were compared*.* Compared to Veterans who did not have any social work encounters over the year, the Veterans who engaged at least once with a PACT social worker were more than twice as likely to have had an emergency department (ED) visit in the month prior to their social work or primary care encounter (12% vs. 5%) and nearly three times as likely to have had a hospitalization in the previous year (8% vs. 2.5%). In terms of health conditions, Veterans who engaged with social work were more than five times as likely to have dementia (8.3% vs. 1.7%) and 18% more likely to be diagnosed with a psychiatric condition (43% vs. 33%) and 48% to have a substance use disorder (12.5% vs. 8.4%). 9.2% of Veterans who engaged with social work were Black/African American, vs. 7.7% of those who did not. (All differences noted here were statistically significant with *p* < 0.001.)

**Table 1:** Characteristics of veterans with and without social work encounters in fiscal year 2022.

	Veterans with primary care	Veterans with no PACT social work encounter at participating sites	Veterans at participating sites with 1 + social work encounters
No. veterans	507,329 (100.00%)	463,609 (100.00%)	43,720 (100.00%)
One or more social work encounters	43,720 (8.62%)	0 (0.00%)	43,720 (100.00%)
Number of social work encounters	0.15 (0.75)	0 (0.00%)	1.79 (1.91)
Age	64.76 (15.92)	64.41 (15.89)	68.52 (15.75)
Over age 75	139,358 (27.47%)	123,398 (26.62%)	15,960 (36.51%)
Male	475,440 (93.71%)	434,732 (93.77%)	40,708 (93.11%)
Female	31,889 (6.29%)	28,877 (6.23%)	3,012 (6.89%)
Race			
Asian or Pacific Islander	2,860 (0.56%)	2,656 (0.57%)	204 (0.47%)
Black	39,645 (7.81%)	35,616 (7.68%)	4,029 (9.22%)
Other race	10,213 (2.01%)	9,276 (2.00%)	937 (2.14%)
White	418,109 (82.41%)	382,719 (82.55%)	35,390 (80.95%)
Rural or highly rural	315,623 (62.21%)	289,053 (62.35%)	26,570 (60.77%)
Enrollment group: >70% services-connected disability	194,110 (38.26%)	177,240 (38.23%)	16,870 (38.59%)
Hypertension	279,758 (55.14%)	254,753 (54.95%)	25,005 (57.19%)
Congestive heart failure	27,655 (5.45%)	22,433 (4.84%)	5,222 (11.94%)
Diabetes mellitus	123,621 (24.37%)	110,651 (23.87%)	12,970 (29.67%)
Tumor	36,304 (7.16%)	32,059 (6.92%)	4,245 (9.71%)
Homeless or unstably housed	11,648 (2.30%)	8,075 (1.74%)	3,573 (8.17%)
Dementia	11,252 (2.22%)	7,634 (1.65%)	3,618 (8.28%)
Psychiatric diagnosis	173,829 (34.26%)	155,126 (33.46%)	18,703 (42.78%)
Substance use disorder	44,540 (8.78%)	39,086 (8.43%)	5,454 (12.47%)
Elixhauser index	2.45 (2.13)	2.36 (2.03)	3.45 (2.80)
Current smoker	15,887 (3.13%)	13,624 (2.94%)	2,263 (5.18%)
CAN score >95	19,612 (3.87%)	13,861 (2.99%)	5,751 (13.15%)
Acute hospital admission, Previous 30 days	3,183 (0.63%)	2,172 (0.47%)	1,011 (2.31%)
Acute hospital admission, previous 12 months	15,153 (2.99%)	11,812 (2.55%)	3,341 (7.64%)
Emergency department use, previous 30 days	29,935 (5.90%)	24,594 (5.30%)	5,341 (12.22%)
Emergency department use, previous 12 months	125,945 (24.83%)	109,093 (23.53%)	16,852 (38.55%)

CAN, care assessment needs score, a percentile rank of the risk of hospital admission or mortality.

### 3.2. Effectiveness

In a previous study, the impact of program participation on acute hospital admissions was examined and there was a 4.4% decrease in ED use and 3.3% decrease in hospital days associated with additional PACT social work staff (11).

Evidence supports palliative care as an intervention that can improve patients' quality of life, decrease unnecessary costs and increase the well-being of caregivers (14). A previous study examined the effect of the staffing program on the use of palliative and hospice services among Veterans with a recent hospital stay. It found that in this group, the percentage of individuals who used palliative care more than doubled at sites after initiating the PACT staffing program (12).

### 3.3. Adoption

**Figure 1:**
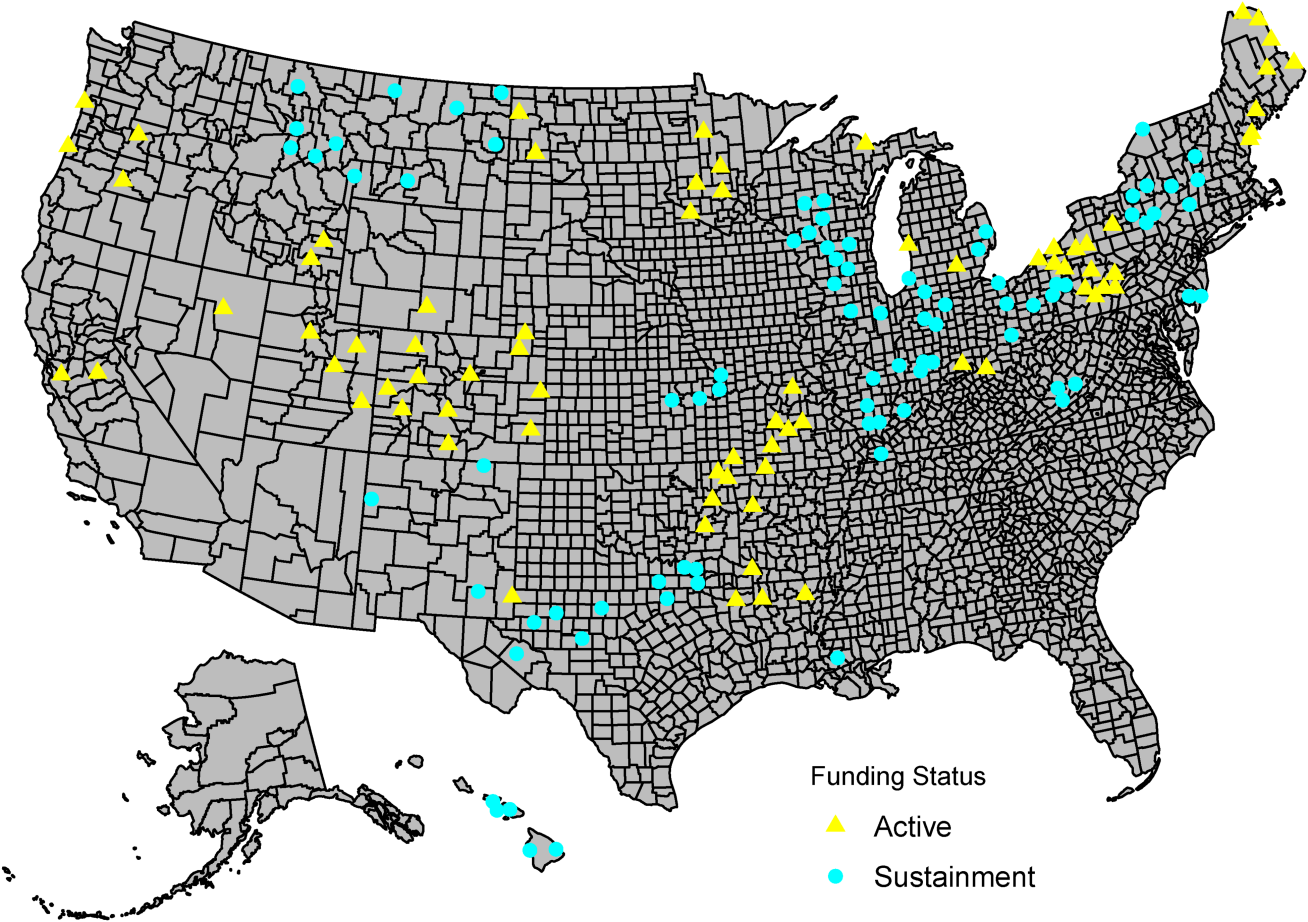
Social work PACT staffing program sites in fiscal year 2022. U.S. map showing all sites participating in the PACT social work staffing program as of fiscal year 2022, in active or sustainment phases.

Figure 1 shows the geographic locations of VA medical centers and outpatient clinics that currently or previously participated in the program. In FY 2022, the program funded 21 active sites in Seed or Sprout phases and a total of 64 social work positions. The program provided support to 23 sites and 77 social work positions in Sustainment and Spread phases.

In FY 2022, the program used a National Social Work Staffing Assessment that was completed in FY 2021 to identify sites that had lower staffing levels in PACT social work and engaged with them to determine if they were ready to implement the Staffing Program, paying attention to national geographic gaps for first consideration. Among 1,141 primary care sites in VA (including VA Medical Centers and Community Based Outpatient Clinics), 167 (15%) participated in the program since its inception, as defined by care provided from a PACT social worker funded by the program. Among those 965 that have never been funded, 397 (41%) were potentially eligible for the rural staffing program. The recommended ratio of social work to Veterans is 0.5 FTE social workers per 1,200-Veteran panel. At those rural sites, the mean ratio of Veteran panels (1,200 Veterans) to PACT social workers was 0.18, and 25% had a ratio less than.07 social workers to Veteran panel. These sites were provided support in preparation for applications for funding in FY 2023.

### 3.4. Implementation

#### 3.4.1. Social work comprehensive assessment, triage, and case management notes

A key element of the National PACT Social Work Staffing Program is the VA Social Work Practice Model, which emphasizes the assessment of psychosocial needs and social risk factors using standardized, national note templates. These note templates provide evidence that the key components of the Social Work Practice Model (triage assessments, comprehensive assessments, and case management) have become integrated into routine practice. In FY 2022, all social workers at Seed, Sprout and Sustainment sites created a total of 68,631 standardized notes used in PACT Social Work practice.

#### 3.4.2. Piloting of a screener for social determinants of health

The implementation component of RE-AIM includes adaptations made to interventions and implementation strategies. The VA does not routinely screen for many health-related social needs, which may mean that Veterans who need social work services do not receive appropriate outreach.

Using ongoing program evaluation and process improvement to address implementation, the program piloted the Addressing Circumstances & Offering Resources for Needs (ACORN) screener (15). The model captures information from multiple sources, including data, feedback from pilot sites obtained during regular check-in meetings and individual consultations with pilot site leadership as needed. Successful use of the ACORN with PACT social workers has led to expansion of the initial pilot. In FY 2022 use of the ACORN tool has been incorporated into Emergency Departments, Advance Care Planning via Group Visits, Heart Failure clinics and with Peer Supports to increase the number of touchpoints where Veterans are screened for needs related to social determinants of health. The most frequently identified needs include the Digital Divide (limited internet access), social isolation and transportation. Veterans with positive screens are referred, most often to PACT social workers, for services and follow-up assessments to address needs.

#### 3.4.3. PACT social work dashboard

**Figure 2:**
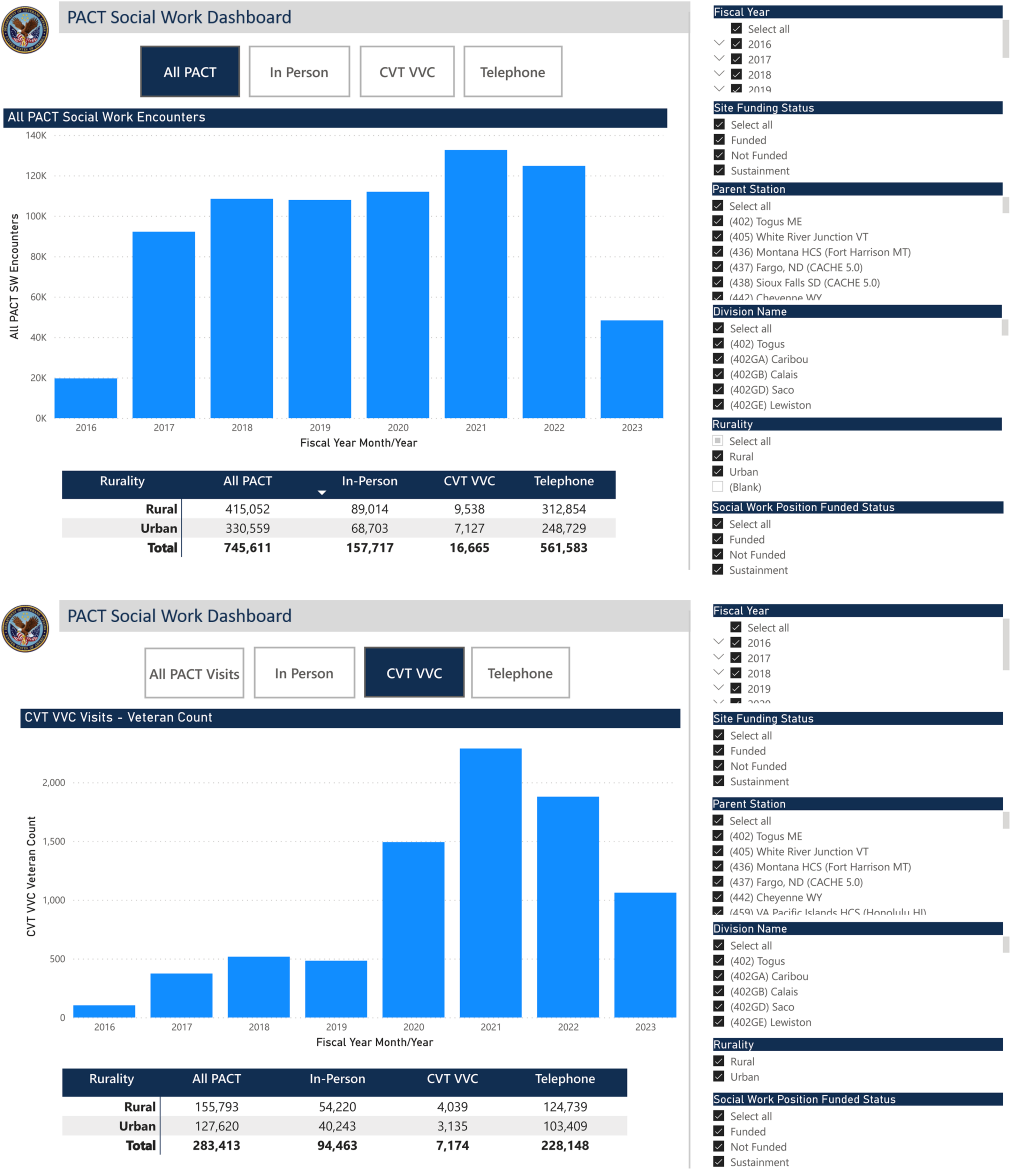
PACT social work dashboard. A report from the PACT social work dashboard. The dashboard is an interactive, visual tool for social workers, champions, supervisors, and program leaders to track program measures. It shows PACT social work encounter and number of veterans with encounters, by fiscal year, month, or quarter, for selected VA stations and clinics, and by rurality and position funding status. The view shown in the figure gives a count of PACT social work encounters provided by all social workers at funded sites.

Previously, social workers relied on monthly spreadsheets to inform them of their site's key evaluation metrics. However, the spreadsheets were not user-friendly. In FY 2021, the team designed and created a working prototype of the PACT Social Work Dashboard (screen shot shown in Figure 2) for sites participating in the Staffing Program to have access to data that is actionable and easy to understand. The dashboard was created using a “human-centered-design” approach, recognizing the users (social workers) as the center of the design process that is also informed by evidence and organizational needs. In FY 2022, user testing was completed with program leaders and site champions and 16 sites participated in a pilot test. The dashboard will enable program leaders, site supervisors and leaders in non-VA organizations to engage in quality improvement by having access to key evaluation metrics and visualizations.

#### 3.4.4. Implementation challenges

A notable barrier to implementation in FY 2022 was continued challenges for recruiting and retaining staff due to impacts from the COVID-19 pandemic. The extensive impact of COVID-19 required PACT social workers to pivot from historically being embedded in a physical location to primarily focusing on the utilization of telehealth and telephone engagement. Challenges unique to rural sites include barriers related to recruitment and hiring of PACT social workers, issues related to the Digital Divide and decreased capacity of community resources such as food banks and transportation services due to increased demand of needs related to the economy and inflation.

### 3.5. Maintenance

Since program inception in 2016, the National Social Work PACT Staffing Program has created 171 social work positions (FTEE) through funding from the Office of Rural Health across 58 sites. It continues to provide support in the form of data and technical assistance to 23 sites in the sustainment phase and 77 permanent social work positions. Since conclusion of funding, 7 sustainment sites have increased PACT social work staffing ratios by adding 16 additional PACT social work FTEE (facility funded), resulting in increased access to social work services and interventions for rural or highly rural Veterans. In addition, in FY 2022, one site invested in 11 new FTEE for PACT social work. This site began with one PACT social worker in the entire state prior to receiving funding for 2 PACT social workers in FY 2020. The impact of the funding on care for rural Veterans led to leadership investing additional resources at the facility level that will provide a great impact to Veterans.

## 4. Discussion

The Social Work PACT Staffing Program found the RE-AIM framework to be a useful tool for planning and real-world evaluation of a national, multi-site program. Program evaluators were able to use the framework as a guideline to design performance measures and benchmarks. However, the framework also had challenges: like previous users, we sometimes had to distinguish the “fuzzy” boundaries among domains and use judgement to decide where a measure should fit (16). Each domain of RE-AIM had advantages and limitations of RE-AIM as a framework applied to this program.

### 4.1. Reach

Our approach to this domain was to compare Veterans who had one or more social work encounters in a fiscal year to those who had none. Veterans who engaged with social work were more likely to have complex care needs, such as a dementia diagnosis or high Care Assessment Needs (CAN) score.

The Staffing Program had challenges in defining a “target population” for the social work program in the spirit of the RE-AIM framework. Tools exist in the VA to identify Veterans with complex care needs. For example, the Care Assessment Needs (CAN) score is a percentile rank of a Veteran's probability of mortality or hospitalization based on their diagnoses and health care use in the previous 12 months (17). But the CAN score and tools like it are designed to predict medical acuity rather than social risk. Defining the target population solely based on medical complexity may risk missing Veterans who experience social needs but who are not (yet) experiencing acute or chronic health problems. Social workers are the clinicians best qualified to identify social needs but even in a fully staffed PACT, social workers do not have the capacity to comprehensively assess every Veteran in a PACT panel. The identification of Veterans in need of social work services necessarily involves educating other members of the PACT clinical team on how to screen for and recognize circumstances where social work intervention may be needed or helpful, referring to social work services when necessary for more in-depth assessment. Social workers can play an important role in educating their colleagues on when to refer Veterans to social work. Team huddles and warm hand-offs can also support appropriate referral practices. Additionally, the ACORN pilot can help identify Veterans who may be in need of social work services.

Universal screening for problems related to social determinants of health, and use of health factors and diagnosis codes related to social needs, are other strategies that will help to define the target population for social work intervention. The Assessing Circumstances and Offering Resources for Needs (ACORN) screening tool is currently being piloted with social work teams and adapted to the social-work clinical settings (15). As use of ACORN is expanded to non-social-work settings, it will create a clinical pathway to identify Veterans with social needs who can benefit from social-work intervention and support.

### 4.2. Effectiveness

Effectiveness refers to the impact of the intervention on individual outcomes, as well as the broader impact of the intervention. In terms of individual outcomes, two studies previously published by the evaluation team showed that increasing social work staffing in PACTs led to improvement in outcomes: decreases in emergency department visits, decreased days in the hospital, and increased use of palliative care services. This domain was of high importance to the staffing program. While social workers use evidence-based practices and refer Veterans to care and resources with established efficacy, prior evidence for the impact of higher social work staffing ratios on patient outcomes was lacking. These studies had a broader impact on the Social Work PACT Staffing Program. These studies, published in high-impact, peer-reviewed journals, were powerful tools for the promotion of social work staffing. Site Champions and Chiefs of Social Work used these articles to make the case to leadership for maintaining the new social work positions or expanding social work staffing.

In evaluating effectiveness, the Social Work PACT Staffing Program contemplated the complex, multi-part nature of the intervention. The staffing program intervention included spark-seed-sprout-sustain stages of capacity-building and technical support for social work services; standardization of social work practice through training and community-of-practice; altering makeup of the PACT teamlet with the addition of social work staff; direction of financial resources to PACTs; and increasing Veterans' access to social work services.

No single measure was appropriate for evaluating the entire intervention due to the program's complexity. Social work intervention at the Veteran level is notoriously difficult to measure. We used the social work “encounter” as one measurable unit of services delivered. While that measure was necessary to measure whether the Staffing Program was delivered, it was not sufficient to gauge whether the program influenced individual outcomes such as emergency department use and whether the program influenced policy. The RE-AIM model was useful for evaluating the Social Work Staffing Program because it is flexible and encourages the evaluation of multiple outcomes, including broader program impacts.

However, the flexibility of the model does present some challenges. For example, many processes fit conceptually under “effectiveness” and “implementation” aspects of RE-AIM. Our approach was to treat the full bundle as an intervention, measuring effectiveness by evaluating staffing levels and healthcare outcomes at the site level. Evaluators and planners of health-system interventions must make decisions about what parts of their program are most important to “unbundle” in order evaluate.

The analytic decision as to when to define the “start” of the intervention also had its challenges. We analyzed the change in outcomes before and after the start date of new social work staff, after “spark” and “seed” stages of the program, which could result in our under-estimating the program's true effect depending on how these earlier stages affected Veterans' care.

A second difficulty common to all evaluations is how to balance pragmatic implementation with study rigor. For understanding the efficacy of person-level treatments, randomized trials with two or more study arms are the gold standard. But the way interventions are studied in controlled settings are often very different from how health care is delivered in the real world. Because a randomized evaluation was not feasible in this context, our team used a quasi-experimental framework that depended on assumptions that our “control group” (sites that had not yet started the staffing program) had trends over time in Veterans' outcomes that the intervention sites would have experienced in the absence of the staffing program. We can try to account for differences in groups that we can measure in VA data, but it is impossible to account for all factors. As RE-AIM continues to develop and be adapted to pragmatic contexts, future work could establish a linkage of RE-AIM to other frameworks for pragmatic impact evaluation when traditional randomized controlled trials are not appropriate or not feasible.

### 4.3. Adoption

Our measure of adoption showed a broad national reach of the program, with some regions that had not yet received funding despite serving majority rural Veteran populations and lacking the full social work staffing ratios. This dimension of RE-AIM provided a useful measure for program leaders to identify areas where they could target outreach for future years of funding. The National Social Work PACT Staffing Program will continue to assess staffing needs across the VA nationally to track adoption of the PACT social work staffing model.

### 4.4. Implementation

Current focus of the staffing program is to focus on use of the National Social Work PACT Staffing Program note templates as a measure of implementation of the Social Work Practice Model. The assessment templates are an important aspect of the model because they provide a guide for social workers to identify Veterans' needs and a standardized way to enter the assessment and plan of care into the Veteran's health record. Relative to the total encounters, the number of notes created would suggest a high rate of use of the templates. High rate of template use is an important measure of fidelity to the intervention, which important to the RE-AIM construct of Implementation. Without measures of fidelity, differences in effectiveness (or lack thereof) could be related to something different from the program being evaluated.

One limitation is that this aggregate measure does not capture potential variation among sites in implementation of the model and could represent an average across sites with high rates of template use and low rates of template use. The program could, in future evaluations, examine variation among sites, and over time, in use of the national templates.

Quantitative implementation data could be complemented with qualitative inquiry into the facilitators and barriers to use of the templates, piloting of the ACORN screener, and the PACT Social Work Dashboard. Qualitative activities had originally been planned in 2020 and 2021 but were cancelled and re-designed due to the COVID-19 pandemic. In future work, for example, the team could use the Practical, Robust, Implementation, and Sustainability Model (PRISM) to inform qualitative data collection, which was developed to complement the RE-AIM framework (18).

### 4.5. Maintenance

The maintenance measure used for this evaluation focused on an institutional level of maintenance: whether funded positions were continued after the end of external funding from the Office of Rural Health. This measure was an important indicator of buy-in from medical center leadership, and demonstration of the need of the position. Future analyses could go further to examine not only whether positions are kept open, but also whether trends in staffing ratios and rates of Veteran engagement with social work continue on similar trajectories in the “Sprout” and “Sustainment” phases of the program.

## 5. Conclusion

Overall, the evaluation team for the National Social Work PACT Staffing Program found that RE-AIM gives a common reference point and a standard of evaluation practice. As we discovered, no one aspect of RE-AIM is sufficient for multifaceted interventions, making the use of the entire framework beneficial. Reach was necessary to assess, as a key program goal was to provide services to rural Veterans. Adoption was used to determine potential sites that were eligible for the intervention. Multiple effectiveness measures were helped us to understand how the program influenced Veterans access to social work and health outcomes. Implementation is critical to gauge fidelity and to create adaptations to changing program demands such as a need to provide telehealth services during the COVID-19 pandemic. Finally, maintenance is needed to understand how to sustain program improvements after funding ends.

## Data Availability

The datasets presented in this article are not readily available because data are protected health information and not available for public use. Requests to access the datasets should be directed to christopher.halladay@va.gov.

## Appendix

**Table A1:** Stop codes identifying social work encounters.

Type of encounter	
In-person	Primary: 323,322,350
	Secondary: 125
	Primary: 125
	Secondary: 323,350,720,707,714
Telephone telehealth	Primary: 147,338
	Secondary: 125
Video telehealth	Primary: 125
	Secondary: 179, 690
